# RegulationSpotter: annotation and interpretation of extratranscriptic DNA variants

**DOI:** 10.1093/nar/gkz327

**Published:** 2019-05-20

**Authors:** Jana Marie Schwarz, Daniela Hombach, Sebastian Köhler, David N Cooper, Markus Schuelke, Dominik Seelow

**Affiliations:** 1 Department of Neuropediatrics, Charité - Universitätsmedizin Berlin, corporate member of Freie Universität Berlin, Humboldt-Universität zu Berlin, and Berlin Institute of Health (BIH), Berlin, Germany; 2 Centrum für Therapieforschung, Charité - Universitätsmedizin Berlin, corporate member of Freie Universität Berlin, Humboldt-Universität zu Berlin, and Berlin Institute of Health (BIH), Berlin, Germany; 3 NeuroCure Cluster of Excellence and NeuroCure Clinical Research Center, Charité - Universitätsmedizin Berlin, corporate member of Freie Universität Berlin, Humboldt-Universität zu Berlin, and Berlin Institute of Health (BIH), Berlin, Germany; 4 Berlin Institute of Health (BIH), Berlin, Germany; 5 Einstein Center for Digital Future, Berlin, Germany; 6 Institute of Medical Genetics, Cardiff University, Cardiff, UK

## Abstract

RegulationSpotter is a web-based tool for the user-friendly annotation and interpretation of DNA variants located outside of protein-coding transcripts (*extratranscriptic* variants). It is designed for clinicians and researchers who wish to assess the potential impact of the considerable number of non-coding variants found in Whole Genome Sequencing runs. It annotates individual variants with underlying regulatory features in an intuitive way by assessing over 100 genome-wide annotations. Additionally, it calculates a score, which reflects the regulatory potential of the variant region. Its dichotomous classifications, ‘functional’ or ‘non-functional’, and a human-readable presentation of the underlying evidence allow a biologically meaningful interpretation of the score. The output shows key aspects of every variant and allows rapid access to more detailed information about its possible role in gene regulation. RegulationSpotter can either analyse single variants or complete VCF files. Variants located within protein-coding transcripts are automatically assessed by MutationTaster as well as by RegulationSpotter to account for possible intragenic regulatory effects. RegulationSpotter offers the possibility of using phenotypic data to focus on known disease genes or genomic elements interacting with them. RegulationSpotter is freely available at https://www.regulationspotter.org.

## INTRODUCTION

In the general search for disease mutations, Whole Genome Sequencing (WGS) is steadily gaining ground. In contrast to Whole Exome Sequencing (WES), it also detects variants within promoters and enhancers, while reducing enrichment problems and artefacts (1). However, the nature and sheer number of variants discovered by WGS pose new challenges for the identification of causal mutations. Whilst the prediction of the effect of variants leading to amino acid substitutions is now relatively straightforward, non-coding variants are much harder to classify. At present, several prediction tools such as GWAVA (2), CADD (3), deepSEA (4), or the REMM score of Genomiser (5) are able to assess ‘extratranscriptic’ variants located outside of transcripts. A recent publication by Rojano and colleagues summarises currently available prediction tools with their advantages and limitations (6). One major drawback of these programs is that they provide results in the form of scores instead of biologically meaningful annotations that are critical for our target audience, clinicians and life scientists. This is inherently problematic since the expertise of the latter groups is indispensable for the determination of the molecular cause of inherited diseases (7,8). A recent study by Shyr *et al.* (9) concluded that the ‘successful adoption of a clinical WES/WGS system is heavily dependent on its ability to address the diverse requirements of specialists in distinct healthcare domains’. They thus propose software interfaces specifically tailored to the needs of different professional groups. Most clinicians prefer graphical interfaces and the limiting of the displayed data to those particular features which are most relevant to their questions (9). In addition, these features must be represented in a meaningful, comprehensive fashion, not as a battery of raw scores. Tools such as Ensembl's Variant Effect Predictor (10) (VEP) or RegulomeDB (11) offer a higher degree of human readable annotation, but do not allow dedicated filtering strategies focused on phenotypic features or candidate genes. We have therefore developed RegulationSpotter, a web-based and user-friendly software for the rapid and convenient annotation and analysis of extratranscriptic DNA variants. In a novel approach to analysing regulatory variants, we focus on the human-readable presentation of the underlying biological data combined with an annotation-based score, thereby rendering RegulationSpotter usable to those clinicians and researchers who lack bioinformatics skills but who still strive to make sense of large sequencing data on their own.

## METHODS

### Software implementation and data integration

RegulationSpotter runs on a 48-CPU system with 512 GB RAM under Linux (CentOS 6). All data used by RegulationSpotter are physically integrated and stored in a PostgreSQL 9.5 database. RegulationSpotter program scripts are written in Perl (version 5.10) and run on an Apache 2.2 web server with HTTPS web protocol. All user interfaces are written in HTML with usage of JavaScript functions and were thoroughly tested for the Firefox browser under Linux, MacOS and Microsoft Windows. Additional testing involves Google Chrome and Safari. We employ TORQUE (version 4.2) as our job scheduling system.

### Training data

We set up two different data sets with extratranscriptic variants (SNVs and InDels) to deduce the biological and clinical relevance of integrated regulatory features. The positive data set **(P1)** contains 457 extratranscriptic disease mutations (tag DM) from the Professional version of HGMD (*HGMD Pro®*, build 2018/1) (12) and the Genomiser publication (5). The negative data set **(N1)** comprises 8,000 randomly chosen common polymorphisms from the 1000 Genomes Project (13), all present in the homozygous state in more than 10 individuals. Further information about the generation of the data sets is given in the Supplementary Material.

## RESULTS

### RegulationSpotter is aimed at clinicians and life scientists

In order to address the need for a clinician-friendly software aimed at the analysis of deep sequencing projects, we have developed RegulationSpotter. Our tool is web-based and performs a comprehensive annotation of single base exchanges and short InDels. RegulationSpotter accepts VCF 4.1 files without any size limitation (e.g. complete WGS runs) and provides an in-depth annotation of all variants chosen to be analysed.

Table 1 summarises the functionalities of RegulationSpotter and comparable tools (see Discussion for details).

**Table 1.** Overview of RegulationSpotter core features and comparable tools. (1) Helpful in this context means any effort to prepare and present the results in a structured or graphical way that helps the user to understand the numerous annotations. (2) This means that all types of variants (known, unknown, SNVs, short InDels) found by Whole Genome Sequencing can be submitted and analysed in a single run and without the need for prior processing of the format or the file size. (3) This refers to the possibility to restrict the analysis to variants residing in candidate genes or their associated regulatory elements such as promoters and distant enhancers or silencers. (✓) CADD scores are only available for a limited selection of short InDels

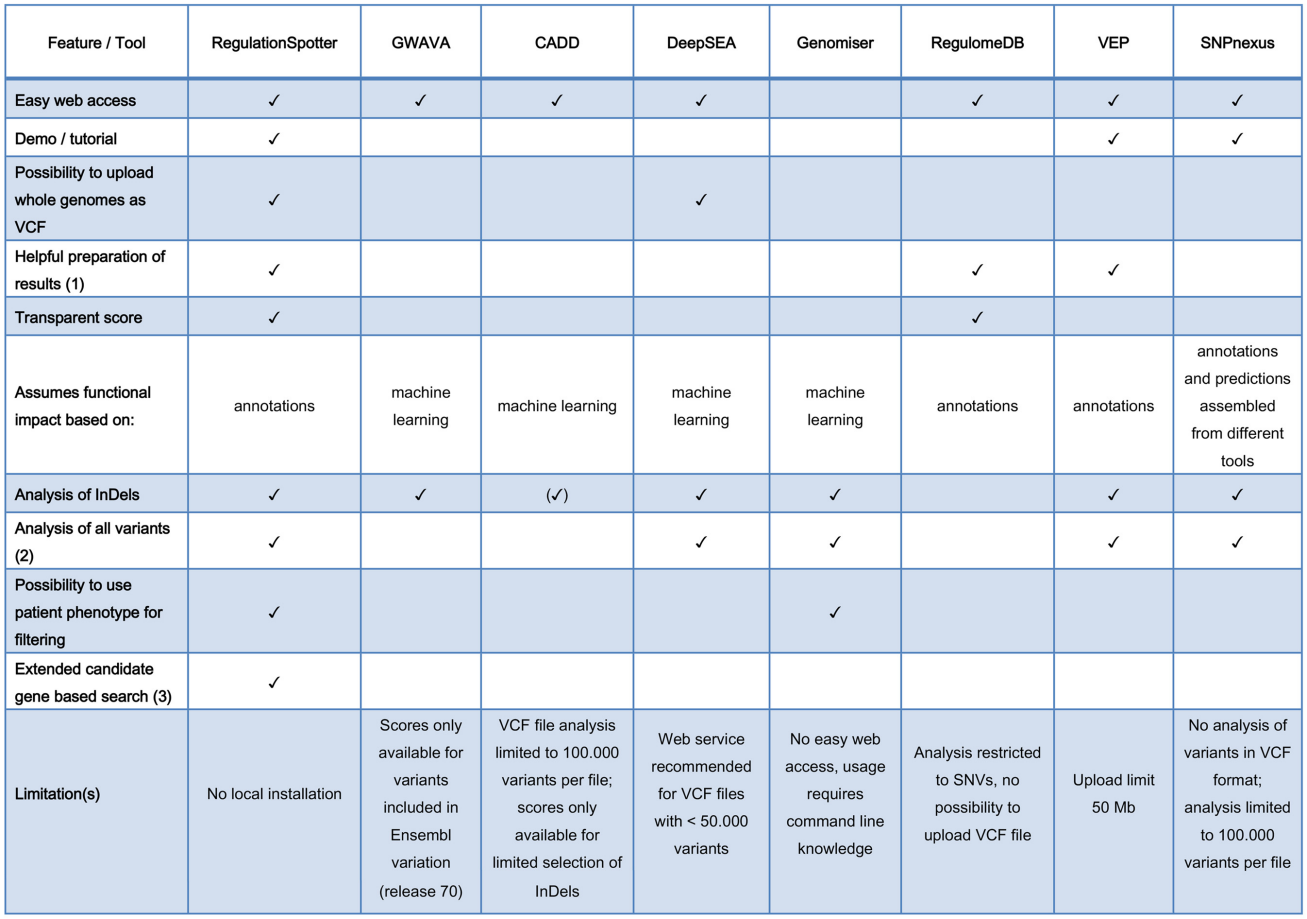

### RegulationSpotter integrates more than 100 distinct genomic features

RegulationSpotter integrates data on gene regulation from different publicly available resources (122 different features) to annotate extratranscriptic variants. We include various tracks from the Ensembl Regulatory Build (14) version 37/91, such as promoters, promoter flanking regions, enhancers, CTCF binding sites, transcription factor binding sites (TFBS) and open chromatin regions. Apart from integrating these precomputed regulatory features, we further processed and merged available annotation tracks to generate customised, refined annotations (e.g. promoters with epigenetic marks which suggest activity detected in at least three cell lines). The Ensembl Regulatory Build also comprises enhancers from VISTA (15) and promoter and TSS (transcription start site) annotations from the FANTOM5 project (16). In order to link distant modifiers such as enhancers to promoters, we use genome-wide interaction data from Hi-C (17) and ENCODE (18,19) 5C (20) experiments and FANTOM5 enhancer-TSS associations analysed with CAGE (21). Moreover, the degree of evolutionary conservation via PhastCons (22) and PhyloP (23) is also included (see Table S1 in the Supplementary Material for details on integrated data).

### RegulationSpotter facilitates the analysis of Whole Genome Sequencing data

**Figure 1.**
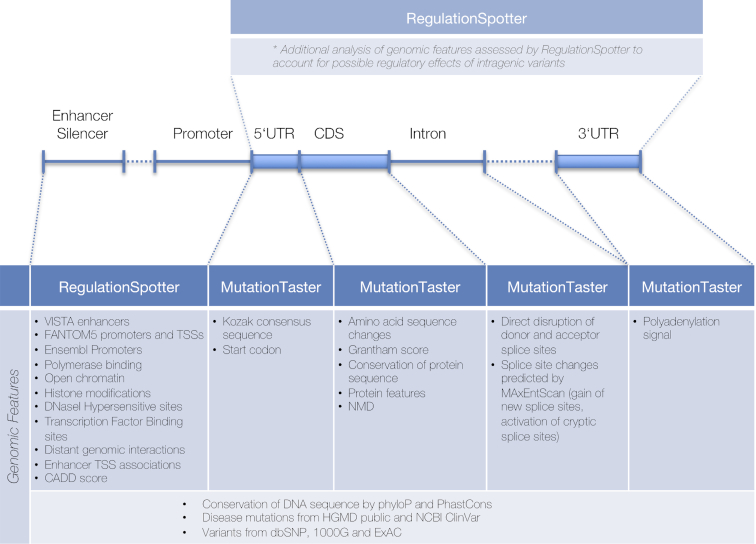
Overview of features that can be assessed in a single RegulationSpotter VCF analysis run. Depending upon a variant's localisation, different aspects are analysed either by RegulationSpotter or MutationTaster. ^(*)^ It should be noted that intragenic variants are always additionally analysed by RegulationSpotter to account for the possible regulatory effects of e.g. non-coding variants. UTR: untranslated region; CDS: coding sequence; TSS: transcription start site; NMD: nonsense-mediated mRNA decay.

RegulationSpotter handles genotype data in VCF format but can also analyse single variants on-the-fly. Single queries are entered via chromosome, position, reference base and altered base. In order to streamline the analysis of data from WGS projects, the software determines for every variant (SNV or InDel), whether it is located within a protein coding transcript or outside, i.e. extratranscriptic. Irrespective of the chosen interface (single variant or VCF file analysis), variants within protein coding transcripts are automatically analysed by MutationTaster (24), our previously published software to predict the disease potential of intragenic variants, as well as by RegulationSpotter, in order to account for a potential intratranscriptic regulatory impact. Extratranscriptic variants are analysed solely by RegulationSpotter. Figure 1 depicts the numerous functional aspects covered by RegulationSpotter and MutationTaster, which can all be assessed in a single analysis run. In VCF file mode, users can initially restrict the analysis to candidate genes or regions, thereby selecting only those variants, which either reside within these genes, their promoter regions, or within interacting *cis*-regulatory regions. In addition, they can choose to exclude variants found in population based databases (currently 1000G and ExAC) with user-specified counts of carriers and/or homozygous carriers. This drastically reduces the run time to about 6–12 hours per genome (depending on server load). Submitted variants (along with the genotype and coverage) and the analysis results are stored in a database. Variants, which have already been analysed, are not re-analysed if uploaded in another project, saving more time. Re-analysis of the same genome, e.g. after a change in the alignment or variant calling pipeline, usually takes less than 10 minutes.

After the analysis, an interface is displayed to select variants, export the data or delete a project (see Supplementary Figure S2). It also gives information on the number of variants that were processed and provides access to the variants excluded. Results can then be downloaded as simple text files, or watched directly online.

To reduce the number of variants to be further inspected, users can easily narrow them down to those located in user-defined genomic regions or affecting candidate genes. These candidate genes can either be entered manually or suggested by RegulationSpotter if the user enters the patient's phenotype, as clinical diagnoses (*via* OMIM (25) or OrphaNet (26)) or by their clinical symptoms (*via* the Human Phenotype Ontology, HPO (27)).

**Figure 2.**
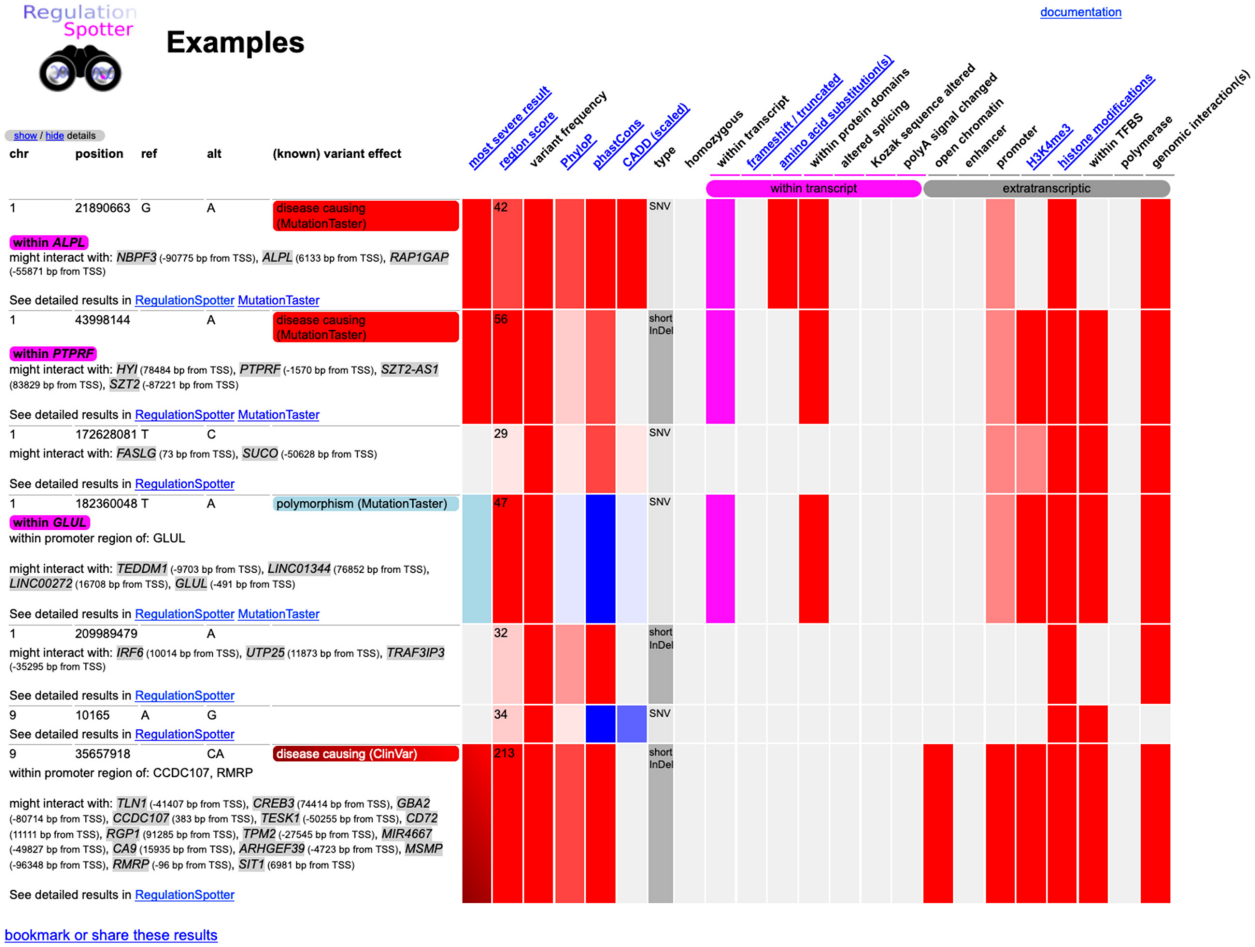
Screenshot of the colour-coded results matrix. Variants chosen to be displayed are organised in a summary table (left part) and in a colour-coded matrix (right part) in order to allow the rapid overview of every variant. Users can follow hyperlinks to study every variant in further detail.

A summary table provides a quick overview of the variants meeting the display criteria (Figure 2 and Supplementary Figure S3). The most relevant functional aspects are displayed in a colour-coded matrix, either as on/off for dichotomic elements (e.g. location within a promoter) or as a colour grade for continuous and discontinuous values (such as conservation or allele frequencies). Hyperlinks guide the user to detailed per-variant results (see Supplementary Figure S4) showing key aspects of every variant and allowing rapid access to more detailed information about its possible role in gene regulation. By grouping different regulatory annotations together by their probable biological role, RegulationSpotter offers intuitive access to the sometimes complex regulatory element landscape. Users can easily generate hyperlinks to RegulationSpotter results, RegulationSpotter can therefore also be employed as a variant visualization tool in other software. Analysis results are stored in our database for at least three weeks, but can also be deleted earlier or stored longer upon the user's request.

### Phenotypic information can help to find the disease mutation

Apart from directly entering candidate genes or regions to filter variants before or after analysis, users can in the variant selection interface also specify a clinical diagnosis, disease (*via* OMIM or Orphanet) or clinical features (*via* HPO) in order to restrict the displayed variants to those residing in disease- or symptom-related candidate genes. The interface offers a text area with autocompletion functionality where entries from OMIM, Orphanet or the HPO automatically pop up while typing in a disease or symptom. Afterwards, a list of associated candidate genes is shown. Users can adjust a threshold to apply a more or less rigid filtering. Variants within distant regulatory regions such as enhancers are also displayed if the regulatory element is linked to a candidate gene. The connection between distant regulatory elements and associated genes is drawn from HiC and 5C data (see Supplementary Material).

### Human-readable evidence for the disease potential of a variant

The results page for a single variant provides details about genomic features present within the region in which the variant is located. Various annotations retrieved from different sources are grouped depending on their assumed role in gene regulation (e.g. promoter and enhancer features), in order to facilitate their interpretation. Hyperlinks to external sources such as the Ensembl Genome Browser (28), the ZENBU genome browser (29) or our web-based tool ePOSSUM (30) (for studying the effects of variants in TFBS) allow quick and easy deeper investigation. Data about genomic interactions are additionally visualised in a plot which shows involved genes or transcripts as well as genomic loci associated with these genes and where on a genomic scale these are located (see Supplementary Figure S5).

Since regulatory information is mostly available for different cell types, we grouped them together into more intuitive colour-coded tissue groups, so that researchers can easily decide if a variant has annotations in their tissue of interest. Although much information is presented, the design allows clinicians and researchers to obtain a rapid yet thorough understanding of a variant's localisation, genomic context, and possible role in regulating disease-relevant genes. By offering annotations from a multitude of different data sources, it saves users from having to manually collect this information on their own.

### Indication of functional relevance of extratranscriptic regions

Apart from annotating variants, RegulationSpotter provides a score (region score), which gauges the functional relevance of the region within which a given variant is located. This score is generated using a subset of 77 features, which showed discriminative power in a testset of known extratranscriptic disease mutations and putatively neutral polymorphisms (see Methods and Supplementary Material for test set assembly and feature selection). In contrast to similar scores generated by other programs, the region score is not generated by a classifier. Instead of applying machine-learning algorithms and risk overfitting to the low number of known extratranscriptic disease mutations, we carried out an initial feature selection and weight determination based on current biological knowledge. We then adjusted the region score on the basis of our preconception as to the effect of the different features and their relative risk ratio of appearing in either a set of 457 extratranscriptic functional variants from HGMD (12) and the Genomiser publication (data set P1) or in 8,000 common polymorphisms from the 1000G (data set N1; see Supplementary Figure S1 for relative risks). This strategy allowed us to limit score-relevant features to those likely to be most meaningful in terms of their biological role (see Supplementary Tables S1 and S2 for exact relative risks and feature weights). To facilitate the interpretation of the region score, we provide a colour-coded translation of the score and the functional relevance of the region. Blue indicates that the region is probably not functional in terms of gene regulation, whereas red encodes likely functional regions. Additionally, we distinguish between poor evidence (pale colour) and strong evidence (strong colour). Knowledge about the functional annotation can help to deduce the relevance of a variant in an intuitive way. However, owing to the relatively small number of known disease mutations that were available for training, we do not assess the effect of the variant itself, only the likely relevance of the genomic region. Intragenic variants, which can be analysed by MutationTaster, are classified as disease causing or polymorphism. Known polymorphisms found in the homozygous state and disease mutations from ClinVar are automatically recognised and classified. The Supplementary Material provides further information on region score generation and integrated features.

## DISCUSSION

RegulationSpotter is a web-based tool for the convenient and streamlined analysis and interpretation of DNA variants from high-throughput sequencing projects. Since it is intended to aid clinicians and life scientists in the interpretation of complex WGS data, our main focus lies on the usability and comprehensive presentation of the results. Our aim has been to offer a broad range of regulatory annotations while also supporting the user in making sense of the presented data. The region score can help in sorting variants according to the amount of regulatory knowledge about the region they are located in. By allowing dynamic post-analysis filtering for candidate genes and regions, researchers can incorporate their clinical or biological knowledge, which is indispensable in the process of identifying likely disease variants. With the combination of (a) offering extensive annotation of complete VCF files from WGS, (b) easily understandable user interfaces and presentation of results and (c) taking into account previous knowledge about biological or disease-related questions, RegulationSpotter stands apart from classical effect prediction tools (see Table 1). The different aspects featured in the table are in our opinion highly relevant for clinicians and life scientists. They help to assess the possible involvement of a regulatory variant in the clinical phenotypes of their patients. For example, a presentation of results that goes beyond the simple listing of annotated features is desirable to facilitate our understanding of the numerous annotations. This can be achieved by graphical display or the grouping of different features in the context of their role in gene regulation. A transparent score facilitates understanding of the assumed functional impact of a variant. The possibility to connect clinical features and diseases to candidate genes, and the subsequent search for variants affecting these, takes into account the clinical expertise of physicians. Simple interfaces and web-based access are crucially important in a clinical setting where software often must not be installed locally. The analysis of all types of variants, going beyond SNVs, guarantees a seamless analysis with reduced effort. Taken together, we consider that RegulationSpotter offers a unique combination of services relevant to our target audience.

We developed the software in an iterative manner and in close collaboration with users, thereby maximising adaption to their needs. By accessing 122 different annotation features from a multitude of sources, RegulationSpotter spares the user from having to collect data on their own and significantly facilitates the in-depth study of potential regulatory variants. Instead of randomly displaying all available annotations, we group the single features together depending on their assumed role in gene regulation. Promoters and enhancers are key regulatory elements in gene expression (reviewed in (31–34)). Typical epigenetic marks for active promoters are trimethylation of H3K4 (H3K4me3) (35) and hypersensitivity to DNaseI (DNaseI hypersensitivity sites, DHS) (36,37), which are both largely invariant across different cell types (37,38). Active enhancers are characterised by monomethylation of H3K4 (H3K4me1) (35), acetylation of H3K27 (H3K27ac) (39) and hypersensitivity to DNaseI (36,37) and are highly specific for different cell types (38). Moreover, these marks have a significant predictive potential in terms of the functional relevance of extratranscriptic DNA sequence variants (40,41). In addition to integrating these precomputed multicell regulatory features from the Ensembl Regulatory build, which are rather broadly annotated due to their cell type-unspecific nature, we highlight H3K4me3 modifications and DHSs overlapping in at least three cell types as relevant custom annotation for active promoters. Enhancers are denoted as either active or poised in certain cell lines / types.

To facilitate further investigations, RegulationSpotter also provides hyperlinks, e.g. to our web-based tool ePOSSUM for studying the effects of variants in TFBS or to our candidate gene search engine GeneDistiller (42). To make maximum use of their own knowledge, users can select diseases and symptoms from OMIM, Orphanet and the HPO to automatically restrict their results to those variants residing in genes or regulatory elements known to cause the disease or phenotype in question. The degree of matching between gene and phenotype does not however affect the scoring. The implementation of a machine-learning algorithm that takes into account phenotypic information is currently hampered by the paucity of solved cases with a known disease-causing mutation and comparably deep and reliable phenotyping. With advances in the area of patient phenotyping and digital health recording, these shortcomings may be overcome in the future.

RegulationSpotter offers annotation and a sorting functionality based on the amount of annotation known for a certain genetic locus. It does not offer prediction of the functional relevance of a variant itself. The low number of functionally validated extratranscriptic disease mutations, most of which affect highly conserved nucleotides, may not truly reflect the majority of extratranscriptic disease mutations. These training mutations mostly exert a strong effect and do not reliably model lesser effects which might become deleterious in synergy with other variants (43). The lack of representative training data is also due to the fact that time- and cost-intensive validation studies such as mouse models are usually only conducted for variants with a clear indication of disease relevance, e.g. deletion of a TFBS or very high conservation. We therefore recommend critically assessing the functional evidence underlying any scores and for this reason offer comprehensive annotations in the first place and only as an addition the region score. We display all the integrated data and the respective sub-scores used to generate it, thereby allowing the user to understand the results without the need for further research on background information. Similar to the output of comparable programs, the region score should not be treated as an absolute criterion for the disease potential of a given variant, but rather should be used as an indicator to prioritize extratranscriptic variants according to their regulatory potential.

As mentioned, RegulationSpotter evaluates variants only based on positional effects, i.e. the available annotation at the locus, rather than considering the nature of the change itself. This is, however, also a common limitation of other available programs. It is known that single base exchanges or small InDels in promoter regions may lead to altered gene expression and ultimately to disease (44,45). However, to our knowledge, there is so far no systematic study, which investigated where, within the promoter, disease mutations preferentially reside.

It remains unclear how the exact nature of a DNA change within enhancer regions interferes with gene regulation. Recent studies in mice addressed the question whether mammalian enhancers typically act in an additive manner or if the regulation of one gene by several enhancers might serve as a kind of redundancy backup (46). Owing to enhancer redundancy, which appears to be a widespread feature of mammalian genomes, deleterious consequences due to changes in only a single enhancer may be prevented. On the other hand, there are so-called ultraconserved enhancers, where variation to just one single enhancer may result in a clear phenotype (47). A recent study raised the point that the still fragmentary knowledge and limited amount of representative training data might currently limit the performance of reliable algorithms that are capable of predicting the outcome of all kinds of regulatory DNA variant (43). More data and deeper annotation are needed for us to be able to deduce general rules about the functional consequences of variants located in distant regulatory regions.

With its focus on user-friendliness and comprehensible presentation of integrated data, RegulationSpotter can help to reduce the number of variants that require follow-up investigation, and we believe that it will become a valuable resource in human genomics research. As new knowledge and more data emerge, we shall be able to continually update and extend the data used by RegulationSpotter and optimise the computation of the region score to offer even better interpretation and annotation of extratranscriptic variants in the future.

## DATA AVAILABILITY

RegulationSpotter is freely available at https://www.regulationspotter.org. No login is required. We provide a thorough documentation along with a tutorial on our website. With simple hyperlinks (position and alleles), RegulationSpotter can easily be used as a downstream application for WGS analysis.

RegulationSpotter accepts single-sample VCF files in VCF 4.1 format as well as single variants in VCF-like notation. Analysis of a WGS project with 3.5 million variants takes ∼4–12 h, depending on the server load. This length of time can be drastically reduced by filtering out common polymorphisms or confining the analysis to candidate genes, their promoters and interacting regions. These options are available in our upload interface. Uploaded data are available only *via* a unique secret URL, which is displayed to our users during the upload process. We strongly recommend to zip large VCF files prior to upload to reduce the upload time, which might be long, depending on the internet speed (e.g. the upload of 1GB at an upload speed of 5 Mbps takes approximately 30 min). The data are automatically deleted from the webserver after 3 weeks unless users actively delete their project or request an extension by E-mail.

## Supplementary Material

gkz327_Supplemental_Files
