# Supplementary material for: RegulationSpotter: annotation and interpretation of extratranscriptic DNA variants
Source: Nucleic Acids Res. 2019 May 20;47(W1):W106–13. doi: 10.1093/nar/gkz327 (PMC6602480; doi:10.1093/nar/gkz327)
Supplement: gkz327_Supplemental_Files [file gkz327_supplemental_files.pdf]

RegulationSpotter: annotation and interpretation of extratranscriptic DNA sequence variants

Supplementary Material

Schwarz, Jana Marie[1,2,3*],Hombach, Daniela[2,3], Köhler, Sebastian[2,4,5], Cooper, David N.[6], Schuelke, Markus[1,3], Seelow, Dominik[2,4]

Charité - Universitätsmedizin Berlin, corporate member of Freie Universität Berlin, Humboldt-Universität zu Berlin, and Berlin Institute of Health (BIH), [1]Department of Neuropediatrics, [2]Centrum für Therapieforschung, [3]NeuroCure Cluster of Excellence and NeuroCure Clinical Research Center; Berlin, Germany

[4]Berlin Institute of Health (BIH), Berlin, Germany

[5]Einstein Center for Digital Future, Berlin, Germany

[6]Institute of Medical Genetics, Cardiff University, Cardiff, United Kingdom

Correspondence should be addressed to:

Jana Marie Schwarz

Department of Neuropediatrics,

Charité – Universitätsmedizin Berlin

Augustenburger Platz 1

13353 Berlin

Germany

Phone: +49 30 450 539 038

Fax: +49 30 450 539 965

Email: jana-marie.schwarz@charite.de

Annotation sources

Ensembl multicell regulatory features

The Ensembl regulatory[1] build assembles epigenetic marks to a genome-wide set of regions that are likely to be involved in gene regulation. The following features can be distinguished and are integrated into RegulationSpotter (genome build GRCh 37 / Ensembl regulatory build version 91):

- **Promoters**
- **Promoter flanking regions**
- **Enhancers**
- **CTCF binding sites**
- **Transcription factor binding sites**
- **Open chromatin regions**

Ensembl regulatory features

Apart from the multicell regulatory features (see above), the Ensembl regulatory build offers all annotation tracks as single features. The following classes are integrated in RegulationSpotter:

- **Histone modifications:** 28 different histone modifications
- **Open chromatin:** DNase I hypersensitivity sites
- **Polymerase binding sites:** Polymerase II and III binding sites
- **Transcription factor binding sites:** 76 different transcription factor binding sites (TFBS)

Enhancer and TSS annotations

We retrieved annotations for enhancers and transcription start sites (TSS) from the FANTOM5 project[2] and the VISTA enhancer browser[3] via the Ensembl regulatory build.

Additional FANTOM5 annotations

We included data on enhancer elements and their interactions with promoters from the FANTOM5 project. Data were downloaded from
http://enhancer.binf.ku.dk/presets/enhancer_tss_associations.bed.

Genomic interaction data

We integrated data on the interaction of distant genomic elements generated by Hi-C experiments from Rao et al.[4], from 5C experiments for the ENCODE project[5,6] generated by groups from the University of Massachusetts and from the 4D Genome database. Data were downloaded from

5C data: https://www.ncbi.nlm.nih.gov/geo/query/acc.cgi?acc=GSE39510

Hi-C data: https://www.ncbi.nlm.nih.gov/geo/query/acc.cgi?acc=GSE63525

4D Genome: https://4dgenome.research.chop.edu/

Phylogenetic conservation

We used the genomic evolutionary conservation scores phyloP[7] and PhastCons[8] derived from multiple alignments of 45 vertebrate genomes to the human genome, downloaded from the UCSC Genome browser from the following URLs:

phyloP: http://hgdownload.cse.ucsc.edu/goldenpath/hg19/phyloP46way/

phastCons: http://hgdownload.cse.ucsc.edu/goldenpath/hg19/phastCons46way/

CADD scores

We retrieved CADD scores for all possible SNVs in the human genome (GRCh37) from http://krishna.gs.washington.edu/download/CADD/v1.3/whole_genome_SNVs.tsv.gz and stored the highest value for each position in our database.

It should be noted that CADD scores are based on similar data than our region score and therefore not used by RegulationSpotter to score a region. CADD scores are integrated in the output as a further information for our users but we recommend to use the hyperlink to their website for a variant-specific analysis.

Human variation

We integrated variants, genotypes and genotype frequencies from the 1000 Genomes Project (1000G)[9] extracted from

ftp://ftp.1000genomes.ebi.ac.uk/vol1/ftp/release/20130502/ALL.autosomes.phase3_shapeit2_mvncall_integrated_v5.20130502.sites.vcf.gz

using tabix and from the Exome Aggregation Consortium (ExAC)[10] version 0.3.

Data sets for training and validation

Positive data sets P1/P2 with functional variants from ClinVar and HGMD

We assume variants in sets P1/P2 ('positive' cases) to be 'functional', i.e. to interfere with gene function or expression.

Training data set (P1) with functional variants from HGMD® Professional

We included 457 variants from the Professional version of the Human Gene Mutation Database (HGMD® Pro, version 2018/1) and the Genomiser publication[11] which are located outside of any protein-coding Ensembl transcript. We confined the variants from HGMD to those tagged with the label **DM** (denoting disease-causing mutations). We also omitted all mutations that were also included the 1000 Genomes Project in homozygous state or in ClinVar[12] release 2018-07-29 (data set P2).

Internal validation data set (P2) with disease mutations from ClinVar

We included 173 variants from ClinVar with CLINSIG codes 4 (likely pathogenic) or 5 (pathogenic) which could not be mapped to any protein-coding Ensembl transcript.

Negative training and validation data sets (N1 and N2) with non-functional variants from the 1000 Genomes Project

Variants present in these data sets are common in the population, which is why we assume them to be benign. Although we cannot rule out functional effects, these should at least be depleted in comparison to the positive data sets P1/P2.

177,396 common polymorphisms located outside of protein-coding transcripts and present in the homozygous state in more than 10 individuals, were randomly chosen from the 1000 Genomes Project data[9] and divided into data sets N1 and N2 (50,000 variants per file). We excluded all variants also found in data sets P1/P2.

Region Score generation and validation

Feature weights, calculation and optimization of the region score

Feature weights and calculation of region score. RegulationSpotter generates a score reflecting the evidence that a variant is located in a functionally relevant region. Each feature is given a specific weight reflecting the assumed impact of the feature. The score represents the sum of the weights

for all features annotated for a given variant. If one feature is annotated multiple times for the same variant, it adds up only once to the score (see Supplementary Table 1 and 2 for features, details on weights and scoring). Owing to the low number of real positive 'functional' training variants, we decided not to employ machine learning approaches, which require a substantial number of training cases. Instead, we opted to base the weights on current knowledge and models about the roles of the different genomic features in gene regulation. The weights are therefore organized as classes describing the features' impact on gene regulation (high, medium, low contribution), each with a different numerical value. By comparing relative risks (see Supplementary Table 1 and Supplementary Figure 1) of appearance of each dichotomous feature in data sets P1 versus N1, we optimized the weights assigned to the respective features. Due to the low number of cases, we decided not to adapt weights to the exact risk differences but to rather move features into another class in case we over- or underestimated their effect. In addition, we chose to regard only features with at least 7/458 occurrences in training set P1 to avoid spurious scoring. 'Rare' transcription factor binding sites are combined in the pseudo-feature 'rare TFBS'. Some features are representative of the same entity (e.g. various promoter annotations from different sources). In such cases, only the single feature with the highest weight is scored.

In order to find optimal weights for the phylogenetic conservation (phyloP and phastCons), we iterated through different combinations of values and selected the model that reached the highest area under the curve for precision/recall. We found that a relatively low contribution of phylogenetic conservation (Supplementary Table S2) to the final score yielded the best performance.

Feature group	Feature	Source	Relative risk	Weight	n (P1)	n (N1)	f (P1)	f (N1)
**CTCF**[1]	CTCF	ECBF	13.3	1	94	787	0.20935	0.01574
	CTCF Binding Site	EMF	1.7	0.1	12	794	0.02673	0.01588
**Open chromatin**[1]	Open Chromatin|DNase1	ECBF	13.8	1	264	2129	0.58797	0.04258
	Open chromatin	EMF	3.9	0.5	26	737	0.05791	0.01474
	DNase1	RS	100.8	10	162	179	0.3608	0.00358
**Histone marks**	H2A.Zac	ECBF	29.4	3	23	87	0.05122	0.00174
	H2AK5ac	ECBF	4.4	0.5	173	4368	0.3853	0.08736
	H2AZ	ECBF	18.9	2	193	1136	0.42984	0.02272
	H2BK120ac	ECBF	5.0	0.5	26	574	0.05791	0.01148
	H2BK12ac	ECBF	3.7	0.2	108	3247	0.24053	0.06494
	H2BK20ac	ECBF	4.5	0.5	14	345	0.03118	0.0069
	H3K14ac	ECBF	4.8	0.5	155	3611	0.34521	0.07222
	H3K18ac	ECBF	5.7	0.5	33	648	0.0735	0.01296
	H3K23ac	ECBF	8.2	1	14	190	0.03118	0.0038
	H3K23me2	ECBF	48.9	5	112	255	0.24944	0.0051
	H3K27ac	ECBF	6.8	0.5	244	4013	0.54343	0.08026
	H3K27me3	ECBF	1.6	0.1	407	28901	0.90646	0.57801
	H3K36me3	ECBF	2.7	0.2	259	10840	0.57684	0.2168
	H3K4ac	ECBF	11.6	1	58	555	0.12918	0.0111
	H3K4me1	ECBF	1.7	0.1	374	24670	0.83296	0.49339
	H3K4me2	ECBF	14.4	1	286	2207	0.63697	0.04414
	RS*H3K4me3[2]	RS	78.0	5	245	350	0.54566	0.007
	H3K4me3[2]	ECBF	26.7	3	282	1175	0.62806	0.0235
	H3K79me2	ECBF	9.0	1	106	1312	0.23608	0.02624
	H3K9ac	ECBF	19.5	2	241	1374	0.53675	0.02748
	H4K20me1	ECBF	1.9	0.2	19	1115	0.04232	0.0223
	H4K5ac	ECBF	14.8	1	61	459	0.13586	0.00918
	H4K8ac	ECBF	9.1	1	117	1426	0.26058	0.02852
	H4K91ac	ECBF	10.9	1	34	346	0.07572	0.00692
**Interactions**	FANTOM5	F5A	15.2	1	18	132	0.04009	0.00264
	HiC	4D	3.9	0.2	235	6742	0.52339	0.13484
**Polymerase marks**	PolII	ECBF	34.2	3	189	615	0.42094	0.0123
**Promoters**[1]	Promoter	EMF	104.3	10	163	174	0.36303	0.00348
	FANTOM TSS (strict)	F5	164.6	20	34	23	0.07572	0.00046
	Andersson promoters	F5A	157.8	20	17	12	0.03786	0.00024
	active promoter	RS	87.6	10	129	164	0.28731	0.00328
	promoter by tss	RS	38.6	3	242	698	0.53898	0.01396
	Promoter Flanking Region	EMF	4.8	0.5	32	749	0.07127	0.01498
**TFBS**	ATF3	ECBF	136.3	10	71	58	0.15813	0.00116
	BCLAF1	ECBF	59.6	5	106	198	0.23608	0.00396
	Brg1	ECBF	40.1	3	9	25	0.02004	0.0005
	Cmyc	ECBF	243.6	20	70	32	0.1559	0.00064
	E2F6	ECBF	139.2	10	85	68	0.18931	0.00136
	Egr1	ECBF	57.9	5	143	275	0.31849	0.0055

ELF1	ECBF	74.7	5	147	219	0.32739	0.00438
ETS1	ECBF	115.7	10	132	127	0.29399	0.00254
FOXA1	ECBF	24.5	2	42	191	0.09354	0.00382
FOXA2	ECBF	39.3	3	30	85	0.06682	0.0017
Gabp	ECBF	46.8	5	61	145	0.13586	0.0029
HDAC2	ECBF	80.0	5	102	142	0.22717	0.00284
HEY1	ECBF	83.8	5	204	271	0.45434	0.00542
HNF4A	ECBF	92.1	5	91	110	0.20267	0.0022
HNF4G	ECBF	102.7	10	95	103	0.21158	0.00206
Ini1	ECBF	71.4	5	50	78	0.11136	0.00156
IRF4	ECBF	139.2	10	70	56	0.1559	0.00112
Jund	ECBF	44.6	5	99	247	0.22049	0.00494
Max	ECBF	98.3	5	83	94	0.18486	0.00188
NFKB	ECBF	41.6	3	75	201	0.16704	0.00402
Nrsf	ECBF	51.3	5	76	165	0.16927	0.0033
p300	ECBF	8.5	1	30	395	0.06682	0.0079
POU2F2	ECBF	76.2	5	132	193	0.29399	0.00386
PU1	ECBF	47.7	5	90	210	0.20045	0.0042
Rad21	ECBF	2.3	0.2	12	576	0.02673	0.01152
Sin3Ak20	ECBF	93.7	5	127	151	0.28285	0.00302
SP1	ECBF	56.6	5	96	189	0.21381	0.00378
Srf	ECBF	39.4	3	29	82	0.06459	0.00164
TAF1	ECBF	71.6	5	220	342	0.48998	0.00684
TAF7	ECBF	45.5	5	40	98	0.08909	0.00196
Tcf12	ECBF	33.3	3	26	87	0.05791	0.00174
USF1	ECBF	48.7	5	109	249	0.24276	0.00498
Yy1	ECBF	60.5	5	151	278	0.3363	0.00556
ZBTB33	ECBF	37.1	3	21	63	0.04677	0.00126
ZBTB7A	ECBF	22.7	2	33	162	0.0735	0.00324
TF binding site	EMF	28.8	3	43	166	0.09577	0.00332
rare TFBS[3]	ECBF/RS	3.6	0.2	56	1736	0.12472	0.03472

Supplementary Table S1: The 75 dichotomous features used to calculate the X-score, along with their relative risk of occurring in the disease mutation group (data set P1/N1). For every variant, every feature is scored only once even if it is annotated multiple times. Sources: EMF = EnsemblMulticellFeatures; ECBF = EnsemblCellBasedFeatures; RS = RegulationSpotter; 4D: 4D data (HiC, 4D, 5C); F5: FANTOM 5; F5A: FANTOM 5 / Anderson

1 Only the feature with the highest weight within this group is scored.

2 If two H3K4me3 annotations are present, only the one with the higher weight is scored.

3 rare TFBS: BAF155, BAF170, BATF, BCL11A, BCL3, BHLHE40, Cfos, Cjun, CTCFL, EBF1, FOSL1, FOSL2, Gata2, HDAC8 Junb, MEF2A, MEF2C, Nanog, Nfe2, NR4A1, Nrf1, Pax5, Pbx3, POU5F1, RXRA, SIX5, SP2, THAP1, Tr4, XRCC4, ZEB1,

Conservation measure	Weight
**phyloP**	10
**phastCons**	10

Supplementary Table S2: Scoring weights for phyloP and phastCons. For each variant, the degree of evolutionary conservation is determined using phyloP and phastCons scores. Both add to the score with their value multiplied by a weighting of 10. PhyloP values are internally normalised to values between 0 and 1.

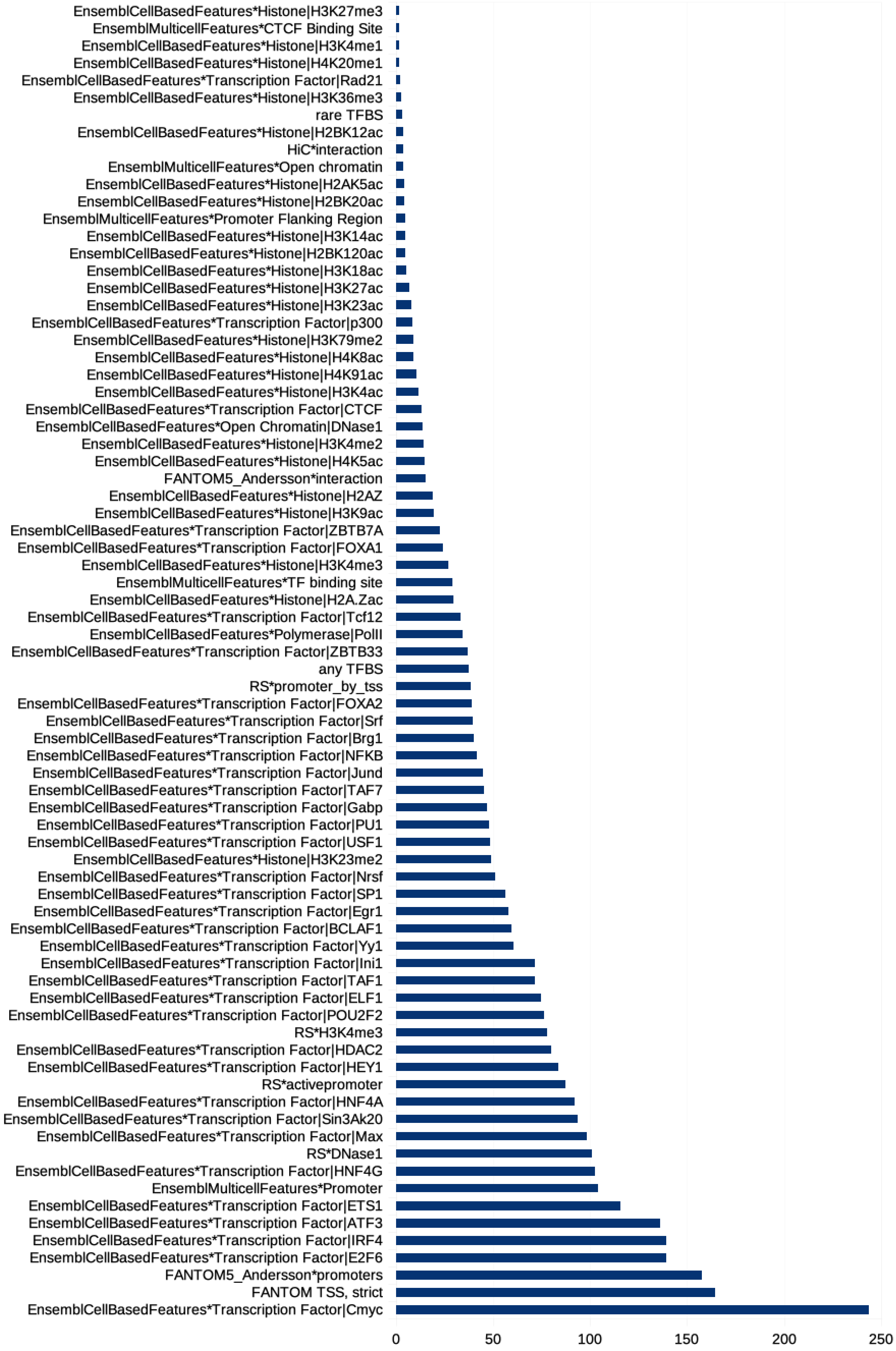
EnsemblCellBasedFeatures*Histone|H3K27me3
EnsemblMulticellFeatures*CTCF Binding Site
EnsemblCellBasedFeatures*Histone|H3K4me1
EnsemblCellBasedFeatures*Histone|H4K20me1
EnsemblCellBasedFeatures*Transcription Factor|Rad21
EnsemblCellBasedFeatures*Histone|H3K36me3
rare TFBS
EnsemblCellBasedFeatures*Histone|H2BK12ac
HiC*interaction
EnsemblMulticellFeatures*Open chromatin
EnsemblCellBasedFeatures*Histone|H2AK5ac
EnsemblCellBasedFeatures*Histone|H2BK20ac
EnsemblMulticellFeatures*Promoter Flanking Region
EnsemblCellBasedFeatures*Histone|H3K14ac
EnsemblCellBasedFeatures*Histone|H2BK120ac
EnsemblCellBasedFeatures*Histone|H3K18ac
EnsemblCellBasedFeatures*Histone|H3K27ac
EnsemblCellBasedFeatures*Histone|H3K23ac
EnsemblCellBasedFeatures*Transcription Factor|p300
EnsemblCellBasedFeatures*Histone|H3K79me2
EnsemblCellBasedFeatures*Histone|H4K8ac
EnsemblCellBasedFeatures*Histone|H4K91ac
EnsemblCellBasedFeatures*Histone|H3K4ac
EnsemblCellBasedFeatures*Transcription Factor|CTCF
EnsemblCellBasedFeatures*Open Chromatin|DNase1
EnsemblCellBasedFeatures*Histone|H3K4me2
EnsemblCellBasedFeatures*Histone|H4K5ac
FANTOM5_Andersson*interaction
EnsemblCellBasedFeatures*Histone|H2AZ
EnsemblCellBasedFeatures*Histone|H3K9ac
EnsemblCellBasedFeatures*Transcription Factor|ZBTB7A
EnsemblCellBasedFeatures*Transcription Factor|FOXA1
EnsemblCellBasedFeatures*Histone|H3K4me3
EnsemblMulticellFeatures*TF binding site
EnsemblCellBasedFeatures*Histone|H2A.Zac
EnsemblCellBasedFeatures*Transcription Factor|Tcf12
EnsemblCellBasedFeatures*Polymerase|PolII
EnsemblCellBasedFeatures*Transcription Factor|ZBTB33
any TFBS
RS*promoter_by_tss
EnsemblCellBasedFeatures*Transcription Factor|FOXA2
EnsemblCellBasedFeatures*Transcription Factor|Srf
EnsemblCellBasedFeatures*Transcription Factor|Brg1
EnsemblCellBasedFeatures*Transcription Factor|NFKB
EnsemblCellBasedFeatures*Transcription Factor|Jund
EnsemblCellBasedFeatures*Transcription Factor|TAF7
EnsemblCellBasedFeatures*Transcription Factor|Gabp
EnsemblCellBasedFeatures*Transcription Factor|PU1
EnsemblCellBasedFeatures*Transcription Factor|USF1
EnsemblCellBasedFeatures*Histone|H3K23me2
EnsemblCellBasedFeatures*Transcription Factor|Nrsf
EnsemblCellBasedFeatures*Transcription Factor|SP1
EnsemblCellBasedFeatures*Transcription Factor|Egr1
EnsemblCellBasedFeatures*Transcription Factor|BCLAF1
EnsemblCellBasedFeatures*Transcription Factor|Yy1
EnsemblCellBasedFeatures*Transcription Factor|Ini1
EnsemblCellBasedFeatures*Transcription Factor|TAF1
EnsemblCellBasedFeatures*Transcription Factor|ELF1
EnsemblCellBasedFeatures*Transcription Factor|POU2F2
RS*H3K4me3
EnsemblCellBasedFeatures*Transcription Factor|HDAC2
EnsemblCellBasedFeatures*Transcription Factor|HEY1
RS*activepromoter
EnsemblCellBasedFeatures*Transcription Factor|HNF4A
EnsemblCellBasedFeatures*Transcription Factor|Sin3Ak20
EnsemblCellBasedFeatures*Transcription Factor|Max
RS*DNase1
EnsemblCellBasedFeatures*Transcription Factor|HNF4G
EnsemblMulticellFeatures*Promoter
EnsemblCellBasedFeatures*Transcription Factor|ETS1
EnsemblCellBasedFeatures*Transcription Factor|ATF3
EnsemblCellBasedFeatures*Transcription Factor|IRF4
EnsemblCellBasedFeatures*Transcription Factor|E2F6
FANTOM5_Andersson*promoters
FANTOM TSS, strict
EnsemblCellBasedFeatures*Transcription Factor|Cmyc
0
50
100
150
200
250

Supplementary Figure S1: Distribution of the relative risks of regulatory features displayed by RegulationSpotter. Relative risks were determined with help of data sets P1 and N1. The text before the asterisk indicates the data source, please see Supplementary Table S1 for details.

To allow a meaningful interpretation of the region score we decided to assess its distribution in a set of known extratranscriptic disease mutations and harmless extratranscriptic variants. In a balanced test set (457 disease mutations from training set P1 plus 457 randomly chosen polymorphisms from N1, we iterated through different region sore thresholds to determine the one which separates the two groups of variants best from each other. We chose the threshold that delivered the highest F1-score to be used to display a simple interpretation of the region score. This can be either or 'non-functional' or 'functional'. To provide further information for our users, we add the label 'much evidence' to the result if the score is above or below the threshold of PPV=98% or NPV=98%, respectively.

In case of available genotypes from 1000G (variant present in homozygous state in more than four individuals) or ClinVar (variant present in ClinVar with CLINSIG code 4 or 5), a variant is automatically denoted as polymorphism (i.e. harmless) or disease-causing. The calculated region score is nevertheless displayed as additional information for the user.

Usage of RegulationSpotter

Analysis of VCF files

RegulationSpotter accepts single-sample VCF files in VCF 4.1 format. Analysis of a WGS project with 3.5 million variants takes approximately 4-12 hours, depending on the server load. This length of time can be drastically reduced by filtering. Adjustable options include the possibility of restricting the analysis to homozygous variants and to set a coverage threshold as well as a frequency filter for variants present in the 1000 Genomes Project (1000G) data[9] and in ExAC[10] (for intratranscriptic variants). Given the huge number of extratranscriptic variants, we suggest limiting the study of variants to those located within a candidate gene, including its promoter region, or in modifiers interacting with that gene.

These options are available in our upload interface. Uploaded data are available only via a unique secret URL, which is displayed to our users during the upload process. We strongly recommend to zip large VCF files prior to upload to reduce the upload time, which might be long, depending on the internet speed (e.g. the upload of 1 GB at an upload speed of 5 Mbps takes approximately 30 minutes). The data are automatically deleted from the webserver after 3 weeks unless users actively delete their project or request an extension by E-mail.

To speed up analyses, a dedicated job scheduling system ensures the analysis of uploaded variants in a highly parallel fashion. Intragenic variants are analysed by MutationTaster and RegulationSpotter, extratranscriptic variants only by the latter. Once finished, the pipeline produces a variant selection interface where users also can display a summary of the number of analysed variants and navigate to the log file to see discarded variants (see Supplementary Figure S2). Users can download analysis results or filter and sort their data to watch them directly online (recommended). The variants meeting the filter criteria are presented in a table, with most relevant intra- and extratranscriptic features also displayed in a colour-coded matrix (see Supplementary Figure S3). Additional information includes the nature of the variant itself, its presence in public databases (1000G, ExAC, ClinVar), the RegulationSpotter region score, CADD score and MutationTaster prediction results (for variants within protein-coding transcripts). The software also provides hyperlinks to the detailed annotation of RegulationSpotter (see Supplementary Figure S4) and MutationTaster (if available) to facilitate further study of every variant's potential effects.

RegulationSpotter is freely available at https://www.regulationspotter.org. No login is required. We provide a thorough documentation along with a tutorial on our website. With simple hyperlinks (position and alleles), RegulationSpotter can easily be used as a downstream application of WGS analysis.

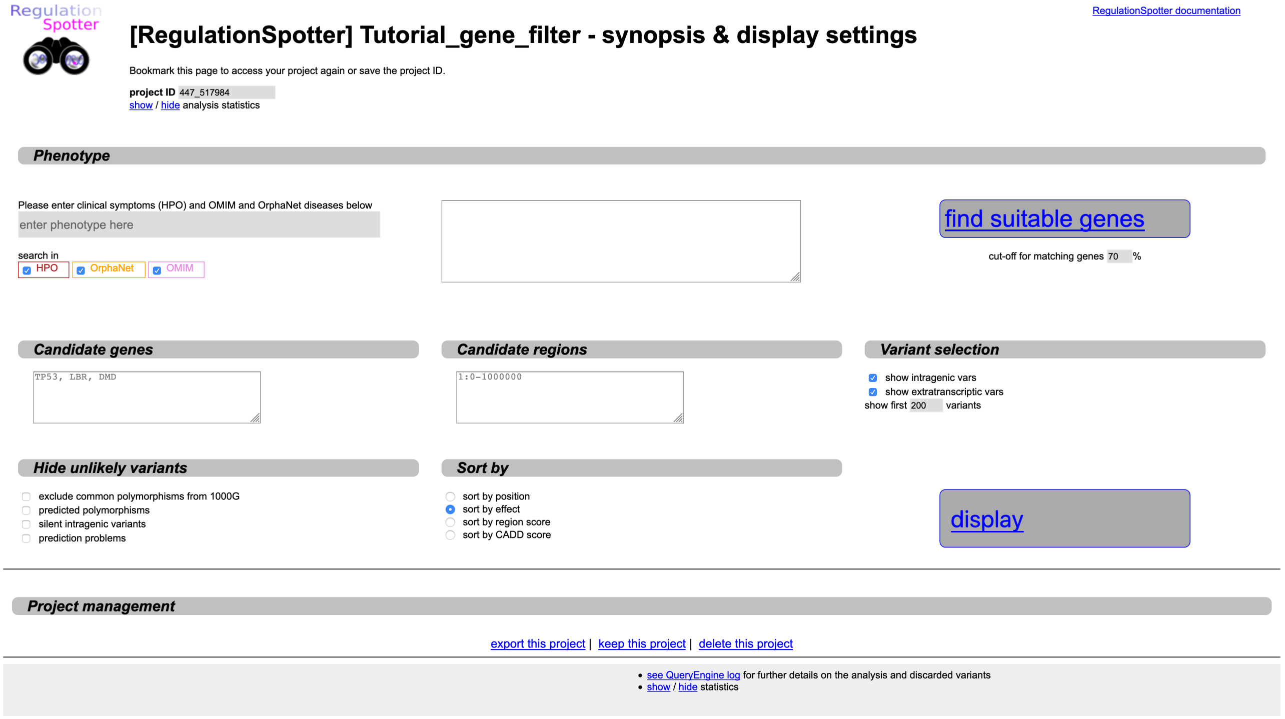

Supplementary Figure S2: Screenshot of RegulationSpotter variant selection page. After uploading a VCF file to RegulationSpotter's QueryEngine, a variant selection page is shown. At the bottom, users can display a summary of the submitted variants and navigate to the QueryEngine log with links to discarded variants. Results can be downloaded or sorted and filtered for watching them directly online.

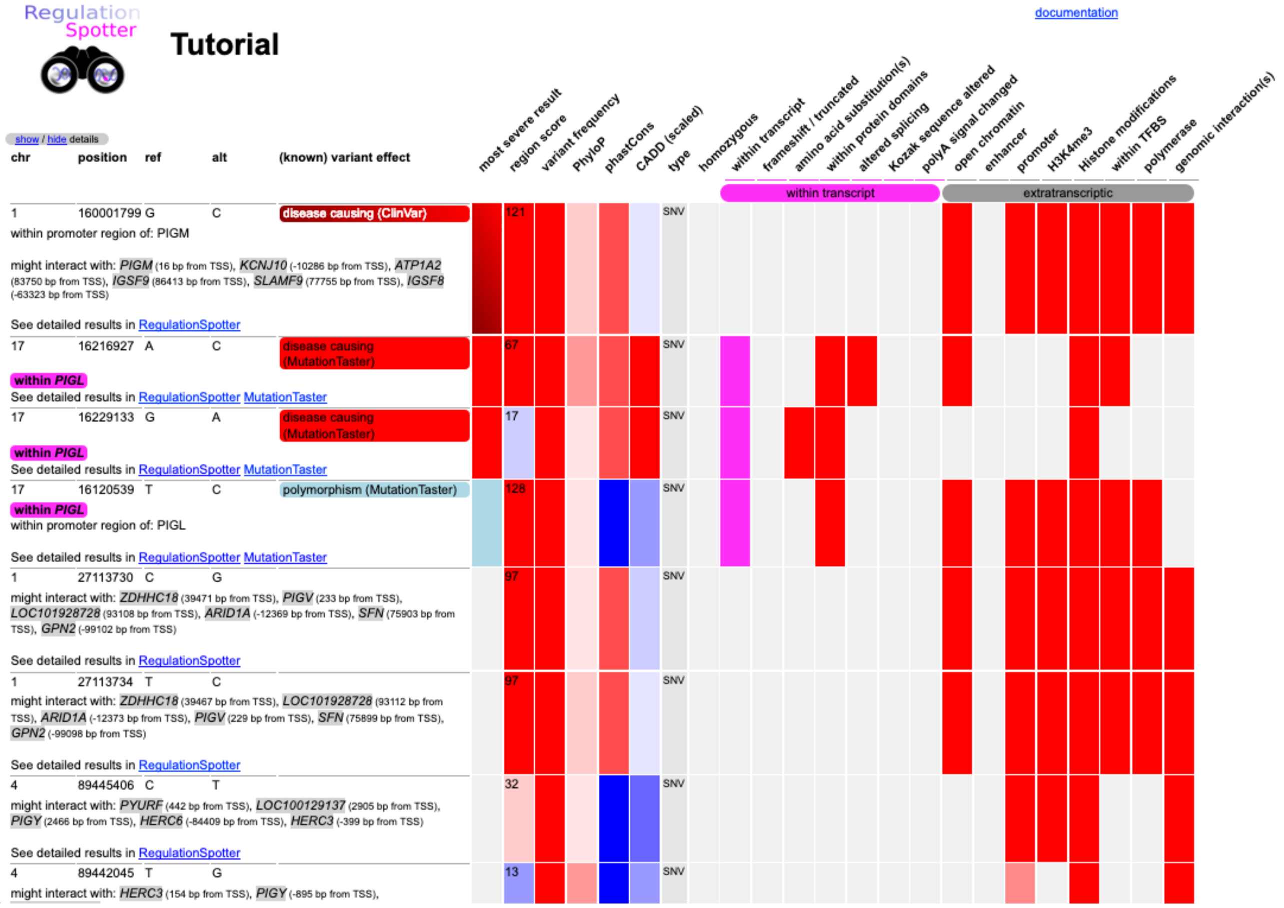

Supplementary Figure S3: Screenshot of the colour-coded results matrix. Variants chosen to be displayed are organised in a summary table (left part) and a colour-coded matrix (right part) in order to allow a quick overview of every variant. Users can follow hyperlinks to study every variant in further detail.

Analysis of single variants

Users can enter single variants by physical position (GRCh37), reference and alternative allele. The single variant results page (see Supplementary Figure S4) contains detailed information about the regulatory features potentially affected by the variant. We group the features by their type, irrespective of their source, but indicate the latter. For every annotation, we offer hyperlinks to detailed explanations in our documentation as well as to the respective data source (e.g. NCBI[13] or Ensembl). We also include hyperlinks to ePOSSUM[14], our tool for TFBS analysis which we did not directly integrate into RegulationSpotter owing to its relatively long processing time. Genome-wide interactions between enhancers and promoters/TSSs are listed in the interface and can be studied in depth in a dedicated graphical interface (Supplementary Figure S5), together with hyperlinks to Ensembl and detailed information about the interacting elements.

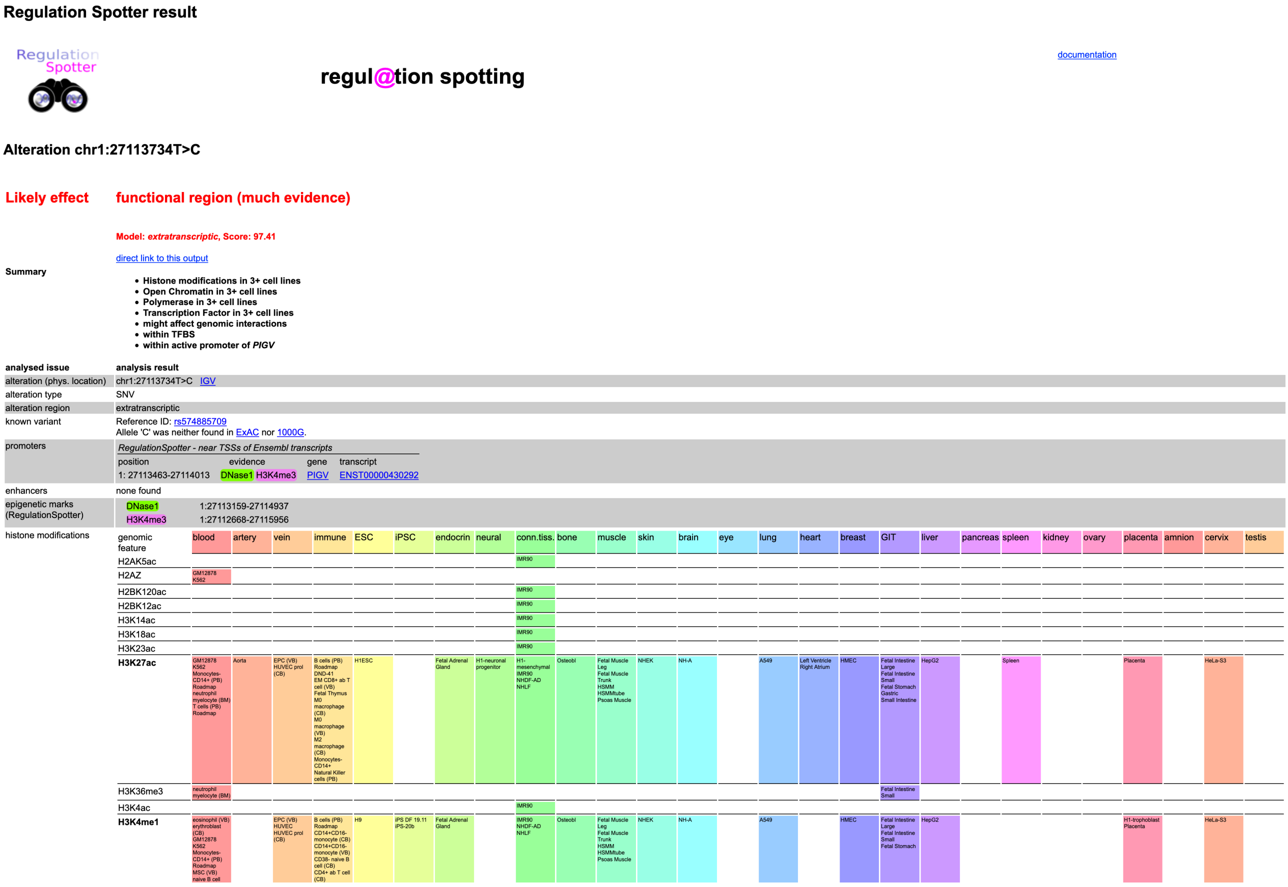

Supplementary Figure S4: Screenshot of a part of RegulationSpotter's detailed results. The detailed output lists all analysis results and annotations that are available for a given variant. Hyperlinks to external resources allow to quickly access additional annotation on the variant and its genomic context.

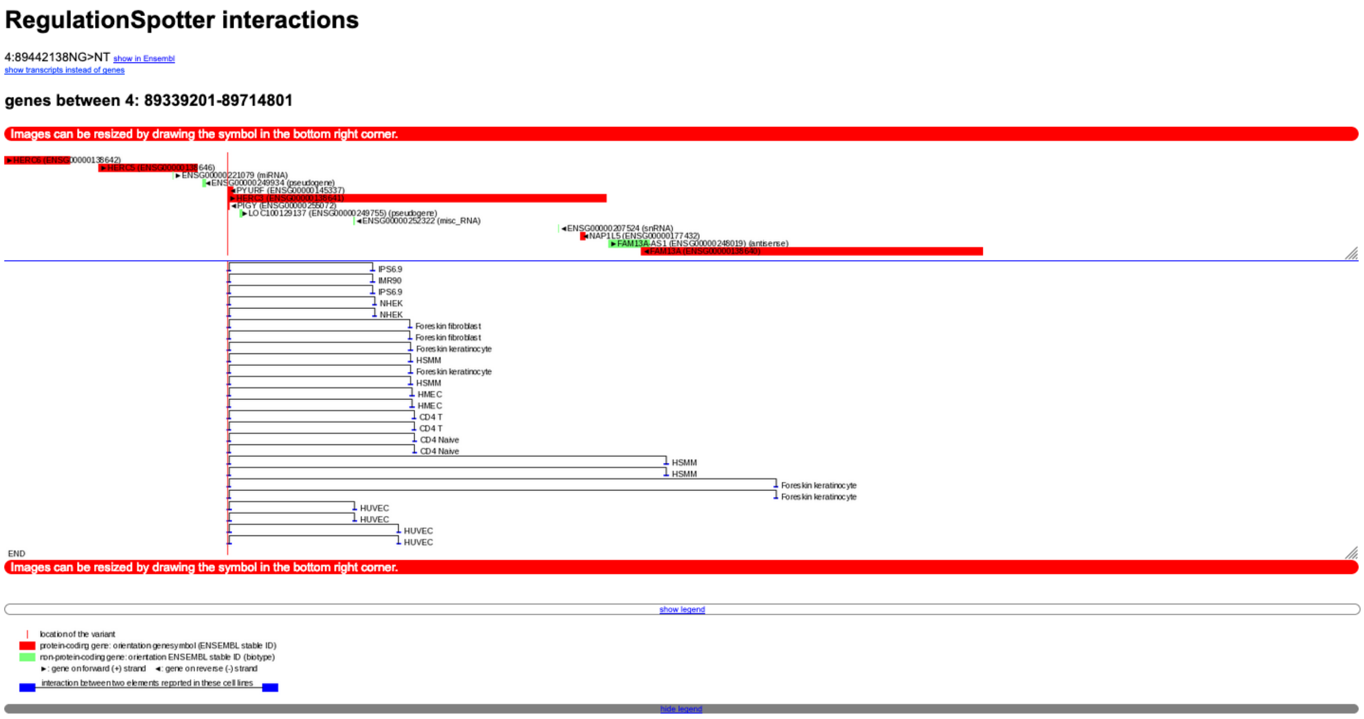

Supplementary Figure S5: Screenshot of the graphical depiction of (distant) genomic interactions.

Implementation

RegulationSpotter runs on a 48-CPU system with 512 GB RAM under Linux (CentOS 6). All data used by RegulationSpotter are physically integrated and stored in a PostgreSQL 9.5 database. RegulationSpotter program scripts are written in Perl (version 5.10) and run on an Apache 2.2 web server with HTTPS web protocol. All user interfaces are written in HTML with usage of JavaScript functions and were thoroughly tested for the Firefox browser under Linux, MacOS and Microsoft Windows. Additional testing involves Google Chrome and Safari. We employ TORQUE (version 4.2) as our job scheduling system.

References

1. Zerbino, D. R., Wilder, S. P., Johnson, N., Juettemann, T. & Flicek, P. R. The Ensembl Regulatory Build. *Genome Biol.* **16,** 56 (2015).

2. FANTOM Consortium and the RIKEN PMI and CLST (DGT) *et al.* A promoter-level mammalian expression atlas. *Nature* **507,** 462–470 (2014).

3. Visel, A., Minovitsky, S., Dubchak, I. & Pennacchio, L. A. VISTA Enhancer Browser--a database of tissue-specific human enhancers. *Nucleic Acids Res.* **35,** D88-92 (2007).

4. Rao, S. S. P. *et al.* A 3D map of the human genome at kilobase resolution reveals principles of chromatin looping. *Cell* **159,** 1665–1680 (2014).

5. ENCODE Project Consortium. An integrated encyclopedia of DNA elements in the human genome. *Nature* **489,** 57–74 (2012).

6. Sloan, C. A. *et al.* ENCODE data at the ENCODE portal. *Nucleic Acids Res.* **44,** D726-732 (2016).

7. Pollard, K. S., Hubisz, M. J., Rosenbloom, K. R. & Siepel, A. Detection of nonneutral substitution rates on mammalian phylogenies. *Genome Res.* **20,** 110–121 (2010).

8. Siepel, A. *et al.* Evolutionarily conserved elements in vertebrate, insect, worm, and yeast genomes. *Genome Res.* **15,** 1034–1050 (2005).

9. The 1000 Genomes Project Consortium. A global reference for human genetic variation. *Nature* **526,** 68–74 (2015).

10. Lek, M. *et al.* Analysis of protein-coding genetic variation in 60,706 humans. *Nature* **536,** 285–291 (2016).

11. Smedley, D. *et al.* A whole-genome analysis framework for effective identification of pathogenic regulatory variants in Mendelian disease. *Am. J. Hum. Genet.* **99,** 595–606 (2016).

12. Landrum, M. J. *et al.* ClinVar: public archive of interpretations of clinically relevant variants. *Nucleic Acids Res.* **44,** D862-868 (2016).

13. Database resources of the National Center for Biotechnology Information. *Nucleic Acids Res.* **44,** D7–D19 (2016).

14. Hombach, D., Schwarz, J. M., Robinson, P. N., Schuelke, M. & Seelow, D. A systematic, large-scale comparison of transcription factor binding site models. *BMC Genomics* **17,** 388 (2016).
